# Integrated analyses of chromatin accessibility and gene expression data for elucidating the transcriptional regulatory mechanisms during early hematopoietic development in mouse

**DOI:** 10.1186/1756-8935-6-S1-P50

**Published:** 2013-03-18

**Authors:** Mahalingam S Viiavabaskar, Nadine Obier, Stella Pearson, Maarten Hoogenkamp, Monika Lichtinger, Georges Lacaud, Valerie Kouskoff, Bertie Gottgens, Constanze Bonifer, David R Westhead

**Affiliations:** 1 School of Molecular and Cellular Biology, University of Leeds, Leeds, LS2 9JT, UK; 2 School of Cancer Sciences, University of Birmingham, Birmingham, B15 2TT, UK; 3 Stem Cell Biology, Paterson Institute for Cancer Research, Manchester, M20 4BX, UK; 4 Cambridge Institute for Medical Research, University of Cambridge, CB2 0XY, UK

## 

The regulation of gene expression is a key factor influencing cell fate decisions during differentiation. The molecular mechanisms underlying gene regulation are multi-layered, with regulatory inputs traversing both within and across different layers, thus providing a very difficult system to decipher. Although the gene expression patterns are cell-type specific, the regulatory inputs follow similar fundamental guidelines. One basic and easily interpretable transcriptional control layer is chromatin accessibility which is a consequence of combinatorial binding of transcription factors (TFs) and various chromatin modifications.

Hematopoiesis is a well-characterized system for understanding transcriptional control mechanisms. The differentiation of hemogenic endothelium (HE) to hematopoietic progenitor cells (HP) forms a crucial step in hematopoiesis as it marks the exclusive commitment of bi-potent stem cells capable of following both hematopoietic and endothelial lineages, to hematopoietic lineages. In this work, we have performed a genome-wide analysis of chromatin accessibility dynamics in the differentiation of HE to HP and subsequently correlated them to gene expression dynamics for comprehending the basic regulatory themes. Chromatin accessibility was examined by global DNasel Hypersensitivity Sites (DHS) mapping during the differentiation, in addition to measuring gene expression by RNA-Seq for HE and HP. Accessible regions unique to HE and HP, and common peaks were identified. In general, there was a direct correlation between the DHS fold change and the expression fold change of the nearest genes for the corresponding peaks, with peaks near promoter regions showing a considerably higher degree of correlation than the rest of the peak populations. In further analyses, we applied a range of bioinformatics approaches such as peak overlap analysis, *de novo* motif discovery, and gene ontology analysis with the ultimate aim to identify the factors influencing lineage commitment. To this end, we have formulated a novel cluster-based approach for studying motif co-occurrence to obtain potential combinatorial TF binding regions, and by correlating those with the expression of the TFs we have systematically explored the relationship between the changes in chromatin accessibility and gene expression dynamics. We are presently developing mathematical models that integrate chromatin accessibility, chromatin modifications and transcription factors to predict gene expression patterns specific for a given cellular differentiation state.

